# The Temporal and Spatial Evolution of Marathons in China from 2010 to 2018

**DOI:** 10.3390/ijerph16245046

**Published:** 2019-12-11

**Authors:** Yifan Zuo, Liye Zou, Mu Zhang, Lee Smith, Lin Yang, Paul D. Loprinzi, Zhanbing Ren

**Affiliations:** 1 School of Management, Jinan University, Guangzhou 510632, China; yifanzuo@stu2019.jnu.edu.cn; 2 Shenzhen Tourism College, Jinan University, Shenzhen 518053, China; zhangmu@jnu.edu.cn; 3 College of Psychology and Sociology, Shenzhen University, Shenzhen 518061, China; liyezou123@gmail.com; 4 Cambridge Centre for Sport and Exercise Sciences, Anglia Ruskin University, Cambridge CB1 1PT, UK; lee.smith@anglia.ac.uk; 5 Cancer Epidemiology and Prevention Research, Alberta Health Services, Calgary, AB T2S 3C3, Canada; lin.yang@ahs.ca; 6 Departments of Oncology and Community Health Sciences, Cumming School of Medicine, University of Calgary, Calgary, AB T2N 4Z6, Canada; 7 Department of Health, Exercise Science and Recreation Management, University of Mississippi, Oxford, MS 38655, USA; pdloprin@olemiss.edu; 8 Department of Physical Education, Shenzhen University, Shenzhen 518061, China

**Keywords:** marathon, spatial distribution, time and space evolution, geographic information systems

## Abstract

The purpose of this study is to explore the spatial distribution pattern and influencing factors of the Chinese marathon. Geographic Information System (GIS) related spatial analysis tools were used to calculate the following—averaged nearest neighbor index, nuclear density analysis and hot spot analysis among others. The spatial distribution evolution characteristics and the influencing factors of eighteen Chinese marathon events in 2010, 129 in 2015 and 342 in 2018 were analyzed. The results show that (a) in 2010 the nearest neighbor ratio was 1.164714, Moran’s I was −0.010165 (type: Random), in 2015 it was 0.502146, Moran’s I was 0.066267 (type: Clustered) and in 2018 it was 0.531149 and Moran’s I was 0.083485 (type: Clustered); (b) in 2010 there was a 333.6 km search radius; the core circle of the Yangtze River Delta was adopted. In 2015 and 2018, a search radius of 556 km was adopted, which was respectively obtained from the core circle of the Yangtze River Delta, the core circle of Beijing-Tianjin-Hebei and the core circle of East China; (c) according to the *Z*-value data, East China and North China in 2015 passed 95% confidence in five provinces and municipal hot spots, passed 90% confidence in three hot spots and passed 95% confidence in Chongqing Cold Point. In 2018, East China, North China, Central Region and eight other provinces and cities’ hot spots passed 95% confidence, four hot spots passed 90% confidence, the Tibet Autonomous Region cold spot passed 90% confidence. Conclusion: The overall distribution of marathon events is greater in the eastern region than the western region, greater in the southern region than the northern region and greater in coastal regions than the inland regions; the nuclear density distribution has spread from the Yangtze River Delta mononuclear circle in 2010 to the core circle of the entire East China region. Moreover, it spread to North China, Central China and South China; the distribution of hot spots spread from Shanghai, Jiangsu and Zhejiang to the entire North China and East China regions. During the past thirty-eight years of development of the Chinese marathon, it has been divided into three stages due to different political, economic and social environments.

## 1. Introduction

### 1.1. Background

As one of the fastest-developing sports in China, marathon running has received much attention from both the public and academia. The first marathon on record in China took place on 17 November 1910, near now Nanjing City, with Jinshan Town, Zhenjiang City as the starting point and Nanjing Nanyang Industrial Exposition memorial tower as the ending point. Although in the early years, marathons in China were held in cities such as Nanjing, Hefei and Beijing, it was not until 1981 that marathons officially started to be developed at a higher level with the opening of the Beijing International Marathon. Eighty-six marathon runners participated in it and twenty-five of them were foreigners. After that, the marathon began to thrive in China and many well-known marathon races have been held, such as the 1996 Shanghai International Marathon, the 2003 Xiamen International Marathon and the 2006 Yangzhou Jianzhen International Half Marathon. In 2010 alone, there were eighteen marathon races held in mainland China. In the following years, the number of events were 22 (2011), 33 (2012), 39 (2013) and 51 (2014). According to the *2018 China Marathon Big Data Analysis Report* published by the Chinese Athletic Association, the size of marathons in China is substantially larger than a decade ago, with 4.98 million participants and 234 cities involved. As shown on www.runchina.org.cn, seventeen marathon races took place on 15 April 2018 in China and if one includes in unregistered marathons, it is estimated that around forty marathon races happened on that day. As the marathon is becoming an increasingly popular event in China, it is important to understand the development of marathons and examine the pattern of temporal and spatial evolution. 

### 1.2. Literature Review

Currently, the marathon is mainly studied from two perspectives. Several studies have considered runners as research subjects and examined the relationship between running a marathon and body functions, as well as the regular patterns [1,2,3,4], psychological impacts [5,6,7] and their cognition [8,9,10,11,12]; while others took competitions as the subject and focused on the sociological aspects, such as entertaining function [13,14,15], fiscal implications [16,17] and marketing and management [18,19]. At present, the studies on Chinese marathons have focused on certain fields, such as physiology [20,21], psychology [22] and sociology [23,24]. Among those, quite a few were focused on the sociological aspects of urban marathons, mainly from perspectives such as the spread of city culture [25], culture coexistence [26], entertainment and tourism [27,28] and sustainability [29,30]. However, little research has been carried out on the temporal and spatial evolution of Chinese marathons [31].

By studying the temporal and spatial evolution of marathon races, we can reveal the developmental pattern of such races in China, examine changes in the political, economic and social environment in China and provide new perspectives for its strategic development in the future. Therefore, to observe the evolution of Chinese marathons from a time-space angle it will aid in the understanding of the development of the marathon in China and its trend of spatial evolution. As the marathon is becoming popular across China, it is necessary to investigate its temporal and spatial evolution, to guide local governments and organizers to better understand marathons and aid in decision-making. Finally, According to the results of the 2017 China Athletics Association’s public data, the number of China’s marathon finishes has risen to fifth place, second only to the United States, the United Kingdom, France and Germany but these developed countries have entered a platform period or a stable period. However, considering China’s population base, government policy support and economic development level, China’s marathon still has great room for development. In the context of the existing international environment, China’s marathon may, possibly, be able to reach the current level of development within five years. Its development history and evolution are more valuable for countries that are developing marathon events.

While in other fields, it is common to see research on the temporal-spatial distribution and evolution of an object. A geographic information systems (GIS) based framework is used to conduct temporal and spatial analysis. For example, GIS has been used to identify the causes of natural or man-made disasters by analyzing the geographic properties of these events, to improve future prediction and preventive measures [32,33]. Likewise, for tourism site development [34] and city transportation system planning [35,36], research has taken advantage of analyzing information on geographic properties to identify the reason or evolution pattern of an object or event and thus to reveal its influential factors or diving mechanism and make predictions, interventions and prevention strategies by referring to its temporal and spatial evolution [37,38,39].

However, the temporal and spatial evolution of the marathon has been rarely studied using GIS. To be specific, only two Chinese researchers, Chen Kunlun, et al. [31] and Ren Jie, et al. [40], have done related research. Both of them studied Chinese marathons which took place in 2017. Chen, et al. (2018) applied the nearest neighbor index, center of gravity model and Kernel density to analyze the spatial distribution and contextual factors of 2017 China high level marathons (The high level events are CAA-certified Class A marathons, which is the highest level of marathon in China.). Their study found that, provincially, marathon events showed a pyramid structure in terms of amount and scale. Spatially, more marathons were held in the eastern and western regions of China than in the inner land; more were held in the south than in the north. The gravity center of marathons was in accordance with that of population density and economy. Based on the findings and previous literature [41,42,43,44,45,46], the researchers concluded that the influential factors for China high level marathons were strong economic strength, better tourism development level, higher social development level and huge population base [31]. Ren (2019) performed average nearest neighbor analysis, Kernel density analysis, buffer analysis, center of gravity analysis and standard deviational ellipse to study the temporal and spatial distribution of marathons in China in 2017. The researchers drew the conclusion that marathons in China were seasonal and most competitions were held during public holidays. Spatially, more marathons were held in the southern coastal areas than in the middle and the west and more were held in the regional centers than in the surroundings [40].

### 1.3. Research Purpose

These two articles set very good examples for future studies but the research subjects were limited, the description was fragmental, the data selected were within the same year, the data of limited types of marathons were visualized, only cross-sectional analysis were conducted, time sequence was neglected, the analysis of China marathons’ temporal and spatial evolution was inadequate and they failed to consider time and space together. The following parameters were also not addressed in the discussed studies—(i) environmental characteristics of cities and regions where the majority of Chinese marathons were held, (ii) distribution characteristics relating to Chinese marathon events between 2010 and 2018, (iii) identification of highlights and hot spots of the Chinese marathon, (iv) stages of which the marathon development can be divided into and (v) from a spatial perspective, the characteristics of each stage. These issues will be addressed in the present study.

Based on previous studies, this paper aims to: use GIS tools to analyze China Marathon data in 2010, 2015 and 2018 and perform quantitative analysis of the expansion and features of China marathons by judging and extracting the information in various locations. Thus, this paper may provide reference and guidance for mid- and long-term marathon planning, site selection and high level competition promotion for marathons in China.

## 2. Materials and Methods

### 2.1. Data Sources

**Figure 1:**
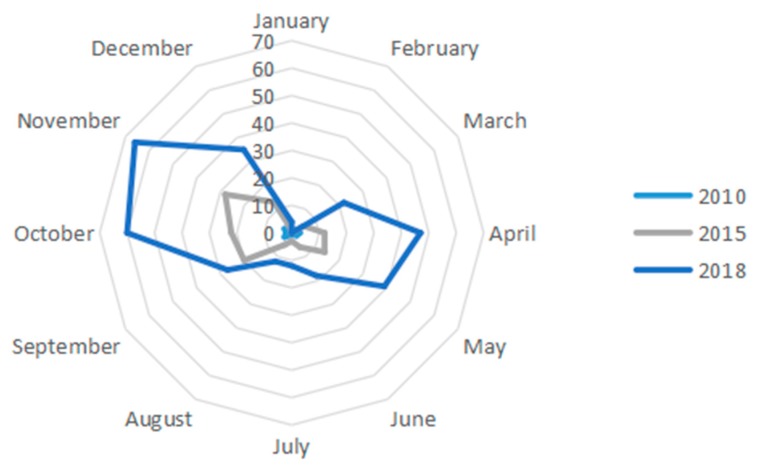
Number of marathons in different years and months. Data source: the official website of China Marathon (http://www.runchina.org.cn/).

First, according to the official website of China marathons (http://www.runchina.org.cn/), there were eighteen marathons held in 2010, 129 in 2015 and 342 in 2018 in mainland China. This number includes various types of marathons (both professional and non-professional). Among them, there were 3 km, mini (around 4 km), 5 km, 6 km, 7.5 km, 8 km, 10 km, family marathon (5–10 km), half marathon (around 21.0975 km) and full marathon (around 42.195 km). The details are shown in Figure 1. 

As can be seen in Figure 1, the number of marathons held in China is increasing yearly. Also, a similar trend can be seen in all three years, which indicates that the seasonal pattern remained consistent. Data suggest that in 2010, an average of 1.5 marathons were held monthly, while in 2015 and 2018, the numbers were 10.75 and 28.5 on average per month, respectively. If we define the months in which more than the average marathons were held as high seasons, then April–May and September–November are high seasons [47,48,49]. 

### 2.2. Research Methods

Spatial analysis is a common type of geographical analysis. Based on the locations and forms of geographic items, it calculates the spatial data with consideration to the property information and extracts the spatial information [50]. GIS is an interdisciplinary application technology which covers iconology, geographic information science and computer science. The database is built based on the attributes of key factors and can be divided into spatial database and time-space database. It analyzes data through efficient multi-source data collaboration and visualization. In this research, ArcGIS10.3 was used to visualize the spatial locations of marathons in China in 2010, 2015 and 2018. The research subjects were prefecture-level divisions. GIS spatial analysis realizes visualization through processing vector data and raster data. All the data collected were processed geographically. Accurate decimal location was recorded by searching on Baidu map (a web mapping service similar to google map, provided by Baidu, Beijing, China). ArcGIS10.3 was used to match the venue of marathons with the geographic locations. The base map was sourced from Chinese Academy of Sciences Resource and Environment Science Data Center. The research center is supported by modern spatial information technology, which realizes the scientific reprocessing and integration of the series of output of China’s resource and environmental science data and the data of relevant national departments. The map has good accuracy and authority. 

#### 2.2.1. Average Nearest Neighbor Index

Spatially, the locations of any geographic features are in one of the three states: clustered, random or dispersed. From the visualized map, we can roughly recognize the type of distribution but the judgement is not accurate. The Average Nearest Neighbor Index (*ANNI*) proposed by Clark and Evans [51] in 1954 is an effective spatial measurement which quantifies the spatial relationships of key points and describes the key spatial points [52]. The ratio of average distance between each marathon and its nearest marathon to the average distance of all marathons in random distribution was calculated as [53]:

(1) $$ANNI=\frac{ANNO}{ANNE}=\sum_{i=1}^{n}\min(d_{ij})\frac{1}{n}/\frac{0.5}{\sqrt{n/A}}$$

In Formula (1), *ANNO* is the average nearest neighbor observation distance, *ANNE* is the average nearest neighbor expectation distance in random distribution, $\min(d_{ij})$ is the distance between any marathon and its nearest marathon, *n* is the total number of marathons and *A* is the total area of region being studied. 

#### 2.2.2. Spatial Autocorrelation (Global Moran’s I)

The spatial autocorrelation index is an important indicator to measure the degree of aggregation of the spatial unit attribute values. According to the Moran’s I index value, *Z* score and *P* value, it is judged whether the overall distribution of Chinese marathon events is agglomerated, discrete or random mode. The calculation formula is:

(2) $$I=\frac{n\sum_{i=1}^{n}\sum_{j=1}^{n}w_{ij}\left(x_{i}-\bar{x}\right)\left(x_{j}-\bar{x}\right)}{\sum_{i=1}^{n}\sum_{j=1}^{n}w_{ij}\sum_{i=1}^{n}{\left(x_{i}-\bar{x}\right)}^{2}}.$$

In Formula (2), *n* is the sample size—the number of spatial positions. *x_i_*, *x_j_* are the observed values of the spatial positions *i* and *j* and *w_ij_* represents the proximity relationship between the spatial positions *i* and *j*. Moran’s I index generally takes a value of [−1,1]. At a significant level, when *I* < 0, it indicates spatial negative correlation, when *I* > 0, it indicates spatial positive correlation and *I* = 0 indicates spatial uncorrelation. The larger the value of *I*, the larger the concentration.

#### 2.2.3. Kernel Density Analysis

Kernel density analysis is used to calculate the density of key elements in their surrounding areas. This method centers on the location of a specific point and distributes the properties of the point within a specified threshold range. The density is the largest at the center and decreases with the distance gradually to zero at the extreme distance. That is to say, the geographic event can happen in any spatial location but the possibility of each location is different. The occurrence possibility is higher in areas with a high point density and vice versa [54]. Select the search radius in the Kernel Density analysis so that it calculates the search radius of the density in the range. Since the output unit is a linear unit based on the output spatial reference projection, the selection of the search radius is particularly important and it can be accurately confirmed only after many experiments.

Kernel density estimation means there are *n* events *S*|*S*_1_, …, *S_i_*, …, *S_n_*|*s* in the research rage R. The density is λ(s) and its estimation is $\tilde{\lambda }(s)$. So the estimation of point density of point *τ* is λ(s) given by:

(3) $$\tilde{\lambda }(s)=\sum_{i=i}^{n}\frac{1}{\tau^{2}}k(\frac{s-s_{i}}{\tau }).$$

In Formula (3), *k* (·) is the Kernel function; *τ* > 0 is the threshold radius; *S* − *S_i_* is the distance between density estimation point *S* and *S_i_* [55].

#### 2.2.4. Hot Spot Analysis

Hot spot analysis (Getis-Ord Gi*) is different from Kernel density analysis. In the hot spot analysis, the partial sum of a certain feature and its neighboring features will be compared with the sum of all the features. When the partial sum is too different from the expectation to become a random result, a statically significant *Z* score will be produced. *Z* score is the number of standard deviations from the mean a data point is. The higher the *Z* score, the denser the hot spot. It can be used to directly recognize the density of Chinese marathons and examine the spatial distribution of hot spots and cold spots. The formula is [56]:

(4) $$G_{i}^{*}(d)=\frac{\sum_{\sum =1}^{n}w_{ij}(d)X_{j}}{\sum_{i=1}^{h}X_{j}}.$$

In Formula (4), $w_{ij}$ is the spatial weight matrix and $X_{j}$ represents the number of national marathon events. In order to facilitate the calculation, it is standardized as:

(5) $$Ζ\left(G_{i}^{*}\right)=\frac{G_{i}^{*}-E(G_{i}^{*})}{\sqrt{V_{ar}(G_{i}^{*})}}.$$

In Formula (5), $E(G_{i}^{*})$ and $V_{ar}(G_{i}^{*})$ are respectively the expected value and variance. $Ζ\left(G_{i}^{*}\right)$ > 0 indicates that the distribution value of region *i* is high and the features are clustered, which makes it a hot spot; $Ζ\left(G_{i}^{*}\right)$ < 0, it indicates that the distribution value of region *i* is low and the features are less clustered, which is called a cold spot. 

## 3. Results

### 3.1. The Temporal and Spatial Evolution of Marathons in China

**Figure 2:**
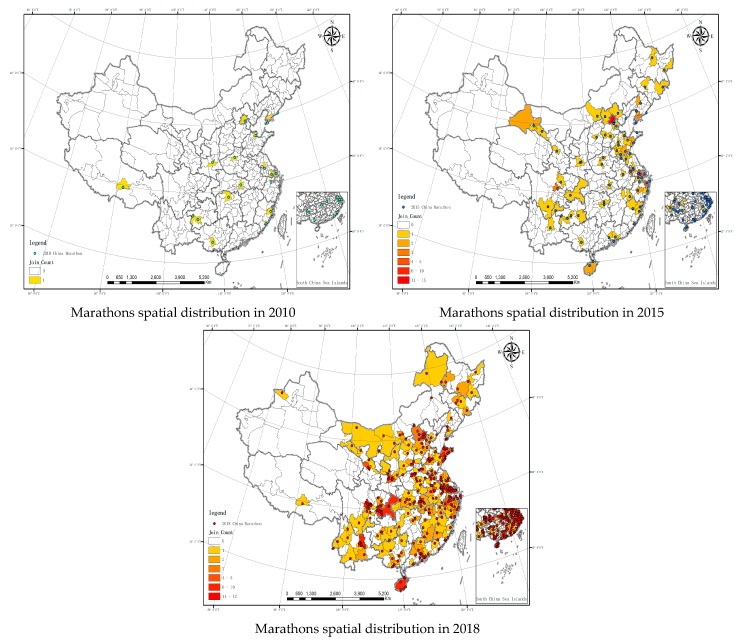
Marathons spatial distribution in 2010, 2015 and 2018. Data source: the Chinese Academy of Sciences Resource and Environment Science Data Center.

The spatial distribution of marathons in China in 2010, 2015 and 2018 was visualized using the Quantities module in ArcGIS 10.3 (Figure 2). 

(1) The spatial distribution of marathons in 2010. Generally, the events were evenly distributed. 18 events were distributed in different cities (prefectures or leagues). Two marathons were held in each of the following province: Jiangsu, Fujian, Guizhou and Liaoning. (2) The spatial distribution of marathons in 2015. With the favorable policies, the number of marathons increased rapidly and more cities started to hold marathons. In 2015, merely in Beijing, fifteen marathons were held, followed by Shanghai, where ten were held. In cities such as Nanjing, Suzhou and Ningbo, five marathons were held. The number of marathons in other areas were similar. Compared to 2010, some inner provinces such as Gansu, Hebei, Inner Mongolia and Jiangxi held their first marathon. In this year, provinces such as Guangdong, Jiangxi and Hebei grew rapidly while there was no marathon held in Tibet. (3) The spatial distribution of marathons in 2018. Except in Qinghai province, all other provinces held marathon events in 2018. All the provincial cities except Xining and Urumqi held marathon events (Table 1). The total number of Chinese marathon events has been increasing year by year, the 2010 marathon events have been scattered and there have been clusters in 2015 and 2018. Overall, the distribution of Chinese marathon events has significant regional differences. There are obvious regional differences in the east, middle, west and northeast. The eastern region is the most distributed, the central region is the second, the western region is the third and the northeast is the least. From the perspective of north-south division, the southern region has an absolute advantage in terms of the number of events and the number of cities where the events are held.

To better compare the density of marathons in 2010, 2015 and 2018, average nearest neighbor was applied to measure the average nearest neighbor distance of the three years. The result is shown in Table 2. The shorter the distance, the stronger the connection. 

It is indicated that, before 2010, the sites of marathons in China were relatively random. In terms of spatial analysis, there was no significance and no regularity. The data in 2015 and 2018 shows that there was not much change. As can be seen from the distance analysis, the distance was five times shorter than that in 2010. The spatial analysis was significant and the features were clustered, which means the connection was stronger.

The spatial autocorrelation analysis was performed for the Chinese marathon events in 2010, 2015 and 2018. The results are shown in Table 3. The Moran’s I indexes of the Chinese marathon events in 2010, 2015 and 2018 were −0.010165, 0.066267, 0.083485, respectively. 2010 was negative, 2015 and 2018 was positive, which indicates that the spatial distribution of the cities hosting the Chinese marathon in 2010 is not relevant and the spatial distribution of the cities hosting the Chinese marathon in 2015 and 2018 are relevant. The *Z* score of China Marathon in 2015 and 2018 are respectively greater than 5.489 and 6.126 and the P values are infinitely close to 0, less than 0.01, has 99% confidence level, the probability of generating a random pattern is less than 1% and the spatial distribution belongs to agglomeration mode, which is consistent with the output of *ANNI*.

### 3.2. Kernel Density Analysis of Marathons in China

**Figure 3:**
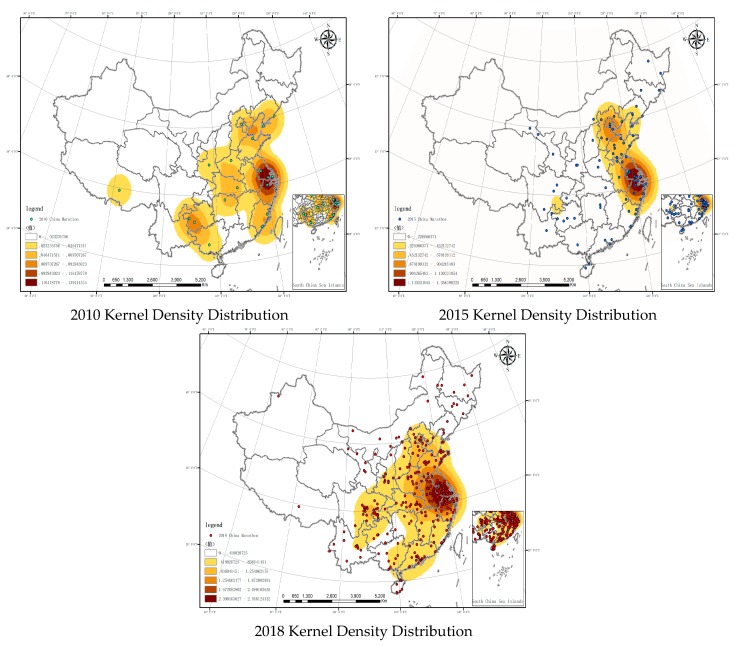
Kernel Density Distribution of marathons in China in 2010, 2015 and 2018. Data source: the Chinese Academy of Sciences Resource and Environment Science Data Center.

In order to demonstrate the spatial differentiation of Chinese marathon tourism more intuitively, the density analysis is used to measure the spatial aggregation area. The Kernel Density tool integrated in the Spatial Analyst in ArcGIS 10.3 is used for Kernel density analysis. After multiple trials, the search radius was 556 km to generate the Kernel density distribution of Chinese marathon tourism in the three years (Figure 3).

(1) Kernel Density Distribution of marathons were dispersed in China in 2010, a small core circle was formed in the Yangtze Delta. To be more specific, the circle was formed by Shanghai, Hangzhou, Yangzhou and Suzhou. (2) In 2015, Kernel Density Distribution of marathons expanded to the surrounding area on the basis of the Yangtze core circle and a sub-core circle was formed among Beijing, Tianjin and Hebei—the Jingjinji core circle. Marathons were clustered in North China to East China regions, mainly in coastal areas. In other inner areas, marathons were still dispersedly distributed. (3) In 2018, The Yangtze Delta area core circle has been expanded to become the East China area core circle and continued to expand to North China, Central China, South China and even Southeast areas. 

### 3.3. Hot Spot Analysis of China Marathons

**Figure 4:**
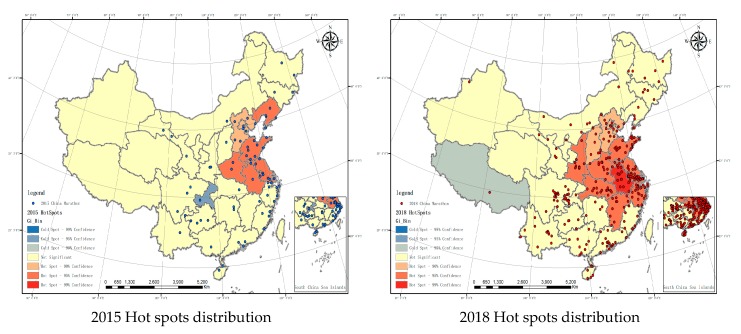
Hot spots distribution of marathons in China in 2015 and 2018. Data source: the Chinese Academy of Sciences Resource and Environment Science Data Center.

In order to reveal the evolution and spatial distribution of hot spots of marathons in China more effectively, the Getis-Ord Gi* index was used to analyze the spatial correlation features among marathon events in 2015 and 2018. This value can be used to identify spatial clustering with significant high value (hot spot) and low value (cold spot) and conceptualize the spatial relations (Figure 4). As the number of marathons in 2010 was under 30, the output would not be reliable, so it was not analyzed here.

All provinces were divided into seven levels. The colors represented the degree of popularity, with deep red representing very popular and dark blue representing very unpopular. The red area has high values and high popularity, so it is a hot spot. See Table 4 for details. (1) Hot spots analysis of marathons in China in 2015. The hot spots and sub-hot spots have been formed in places such as Shanghai, indicating that marathons in the region were many and dense. The marathons held in these regions were more than in the surrounding areas and were clustered. The marathons in this region were rich and diverse and meanwhile it promoted the marathon events in the surrounding areas. However, the *Z* score for Chongqing is relatively low and it was a cold spot. That indicated that Chongqing was isolated in terms of marathons. When the marathon was rapidly development in surrounding areas, Chongqing was left behind. (2) Hot spots analysis of marathons in China in 2018. The scope of the hot spots expanded significantly, with Anhui being the hottest region and its surrounding areas being hot spots. Anhui Province is not an economically developed area in China but marathons were also popular in Anhui. Anhui was positively influenced by the surrounding areas in terms of marathons. There was a sub-cold spot in Tibet, which indicated that more events were held in the surrounding areas of Tibet than in Tibet. From a spatial perspective, marathons in Tibet should be well developed but its natural conditions hindered the development of marathon in Tibet. 

## 4. Discussion

### 4.1. Analysis of the Temporal and Spatial Evolution of Marathons in China

The spatial distribution did not change dramatically from 2010 to 2018. The marathon expanded from its center areas in 2010 to the surrounding areas. The overall pattern was that, if Hu Line was taken as a division, more marathons were held in the east than in the west; if Qinling-Huaihe Line was taken as a division, more marathons were held in the south than in the north and more were held in coastal areas than in the inner land. However, there were some exceptions. For example, in tourism areas such as Sichuan, Yunnan and Chongqing, no less marathons were held than in coastal or east areas. CHEN (2018) mentioned that the distribution of high level marathons in China was uneven, with the east > the west > the middle > the northeast [31]. But if one considers the marathons acknowledged by the official website of Chinese marathons, the distribution should be the east > the middle > the west > the northwest. The reason was that, in 2010, all the marathons held were high level marathons, while with the development and popularity of marathons—as the economic states of the middle was better than that of the west and the population in the middle was larger than that in the west—more ordinary marathons were held in the middle. It is worth mentioning that there are 177 high level marathon events in China in the study of Chen (2018) and 27 provincial administrative regions except Tibet, Xinjiang, Qinghai and Tianjin are distributed. The closest distance to the event is 119.83 km and the nearest index is 0.936632. The distance between all marathon events is closer and the degree of aggregation is tighter. There are some differences in the conclusions of this study.

From the Kernel Density and Hot spot analysis results. It is to some extent common with the distribution of high level marathons. According to the three steps of Chinese terrain, the first step is not suitable for marathons due to the climate and terrain feature and therefore marathons are mainly held in the second and third steps and decrease from coastal areas to the inner land [31]. The reason was that the participants and the urban population increased significantly. Since 2010, the cities with more than 500,000 inflows were mainly in the eastern coastal areas. Among provincial capitals in the middle and western areas, only Chengdu, Xi’an, Wuhan and Zhengzhou reached this number, which coincided with the distribution of the marathons in 2015 and 2018. However, what’s more important was the collective development of city clusters. The development of city clusters has an impact on the cities’ cultural demand, enhances citizens’ manners and qualities, accelerates economic development and promotes social progress [57]. As a national city cluster, the Yangtze Delta city cluster is China’s core economic region. It acts as the main stage for China to participate in international competition and it is the earliest-developed area with the best urbanization. Therefore, before 2010 when the development of marathon was slow in China, many marathons were still held in the Yangtze Delta. Also, during the rapid economic development and policy-assisted growth, the Yangtze Delta area has seized the opportunity to promote the rapid development of the quantity and quality of marathon events to a larger scale and to lead the expansion China urban marathons to other regions.

At present, the holding of a Chinese marathon event can form a multifunctional composite leisure travel space that integrates food, housing, travel, tourism, shopping, entertainment and so forth. The marathon can not only meet the comprehensive and experiential nature of urban leisure space but also it can satisfy the health and culture of urban residents. Taking the 2018 Beijing Marathon (http://www.beijing-marathon.com/) as an example, the leisure space around the Beijing Marathon track has the following characteristics: 42 supply points are set up throughout the marathon track, of which twenty-four are in the Haidian District, 12 are in the Chaoyang District, 5 are in the Xicheng District. and one in Dongcheng District. The total number of leisure places in the four districts is 101 in Haidian District, 58 in Dongcheng District, 50 in Chaoyang District and thirty-eight in Xicheng District. Comparing the number of leisure places around the marathon track, the following quantitative relationship can be observed: Tourist attractions > Museums > Parks > Libraries > Shopping malls > Science Museum > Creative Industry Park > Sports Hall > Art Museum.

### 4.2. Development Characteristics of China Marathon at Various Stages

The spatial expansion and evolution of marathons in China is a dynamic and complex process. The changes in its spatial structure and form are influenced by multiple factors. Since 1981 when the marathon was officially introduced into China, the leading mechanisms influencing the development of marathon events at various stages have been different. Each stage has different ways of expansion and the pattern is different. With favorable policies, economic globalization and regional integration, the influential factors for the spatial expansion of Chinese marathons are also constantly changing and associating. According to the development track of the China Marathon and spatial distribution of marathons at various stages mentioned before, the temporal and spatial evolution of China marathons are analyzed from political, economic and social perspectives and divided into three stages: 

The first stage is from 1981 to 2009. Compared with developed countries, China started late in urban marathons and the popularity of marathons was relatively low. In the first city marathon held in 1981, the Beijing Marathon, only eighty-two runners participated and all of them were male. But this marathon has great historical significance. In the following years, some other cities have started to hold marathons. In this stage, the main purpose of participating a marathon was to compete and achieve good score. Most participants were professional. With the progress of China’s reform and opening up, the living standard of the Chinese people have been greatly improved and people now have higher requirement for well-being and fitness. In 1995, the Outline of National Fitness Program was implemented and marathons were once again boosted. Urban marathons have become one of the most effective way of promoting national fitness. However, according to China Athletics Association, there were only five urban marathons from 1981 to 2000 and only 12 in 2009. At this stage, China just started its reform and opening up. Although the economy has developed rapidly, the political environment and people’s literacy had not reached a level which was suitable for the development of marathons. As an ordinary developing country, China did not have enough capitals and resources for comprehensive and balanced development. Therefore, marathons were only held in a small number of cities with better economic conditions or more resources. The purpose was to integrate and centralize resources, prioritize the development of certain areas and gradually extend the marathons to other regions. Overall, at this stage, the polarization effect was achieved with the political impact of the Outline of National Fitness Program, the economic impact of reform and opening up and the social impact of increased population and improve literacy of people. The polarization effect was attractive to peripheral industries. It gathered peripheral resources and factors of production in the center city to form scale-up development and boost the competitiveness of competitions. From the first marathon in 1981, through the five marathons in 2000, to the twelve marathons in 2009, marathons in China were heading to the polarized stage from the dispersed stage. At this stage, economic factors were the most important, which provide early capital, resource base and population base for marathons’ development. 

The second stage was from 2010 to 2014. After the 2008 Beijing Olympic Games, the concept of national fitness has become more in-depth in China. In 2009, the Chinese government named August 8th as the National Fitness Day. As a sport that has been identified as the most effective way to achieve national fitness, marathons developed rapidly. The tier-1 and tier-2 traditional marathons continued to improving. After long-term marketing and operation, the brand awareness of the competitions was constantly improving. Marathon participants’ increased loyalty to brands. The number of marathons as well as participants in tier-3 and tier-4 cities increased rapidly and the popularity of marathons was greatly improved. The number of events increased, the scope expanded, the form diversified and the influence enlarged. However, the change in quantity had not been large enough to lead to the change in quality. This can be clearly seen in Figure 3. Comparing the changes in the nuclear density coverage area of the 2010 and 2015 China Marathon events, the coverage area of the nuclear density in the 2015 China Marathon has significantly expanded. The evolution effect of China marathons switched from polarization to expansion. The outcome of early polarization helped to popularize marathons in the hinterland of China and promoted its development. After that, the two evolution effects proceed simultaneously and complemented each other. The polarization effect continued to accumulate resources. The expansion effect introduced the growing factors to the peripheral regions, thus promoting the development of the entire region. Social factors became the main force impacting the development of marathons, because at this stage, China’s social form began to shift from “labor” to “leisure”. As a sport originated from social life, marathons’ development also began to be driven by social factors. The changes in social structure and form had an impact on people attitudes towards life and values.

The third stage is from 2015 to now. As the fastest-growing period of marathons in China, 2015 witnessed the boost of marathons. At this time, socialism with Chinese characteristics came to a new era. The publication of many national policies such as Guiding Opinions of the General Office of the State Council on Expediting the Development of the Sports Industry and local policies indicated that the main contradiction in China has changed from “the contradiction between the ever-growing material and cultural needs of the people and the backwardness of social production” to “the contradiction between unbalanced and inadequate development and the people’s ever-growing needs for a better life” [58]. With the political support from the government and as the infrastructure being improved, in 2015, marathons no longer need to be approved or authorized in China. The related department insists on a combination of freedom and restrictions and further strengthened the regulations and standards. Also, the Guiding Opinions of the General Office of the State Council on Expediting the Development of the Sports has raised national fitness as a national strategy and taken strengthening people’s fitness and health as a fundamental aim. Marathons provide platform for the mass to exercise. On this platform, the participants can not only meet their needs for fitness but also gain a happy and unforgettable experience. Meanwhile, China’s economy continues to grow and the construction of cities and city clusters is accelerating. Many cities have chosen marathon as a medium for city promotion. Through holding urban marathons, cities become more well-known. The culture of the region is promoted and many inflows are attracted, which stimulate local consumption. Thus, marathons will bring direct fiscal benefits to the cities in terms of tourism, commerce, transportation and media. 

Overall, the marathon in China has progressed from the expansion stage to rapid growth stage and it is driven by political, economic and social factors all together. As a result, the trend of spatial distribution is featured as “clustered in a small scope and dispersed in a large scope” and “prosperity in multiple areas”. The three factors are equally important. Policies and economic basis has created an environment for the boost of marathons in this stage. For a mature urban marathon, social mechanism factors such as the urban population, the overall quality of the citizens (including their perception of fitness and entertainment), the living standard and social environment are also important.

## 5. Conclusions

By using the data from three years (2010, 2015 and 2018), this research studied the features of spatial distribution and patterns of temporal change of marathons in China. The main conclusions are as follows: (1) In 2010, the Nearest neighbor ratio is 1.164714 (type: Random), Moran’s I is −0.010165, the marathons in China were almost evenly distributed with eighteen events being held in different prefecture-level areas in China. Although dispersed, key spatial points formed a Yangtze Delta area core circle. (2) In 2015, the nearest neighbor ratio is 0.531149 (type: Clustered), Moran’s I is 0.066267, Gansu, Guangdong and other places held their first marathon, In the Yangtze Delta area, a core circle was formed and expanded to the surroundings rapidly. A sub-core circle was formed in the North China region—the Jingjinji circle. East China and North China passed 95% confidence in five provinces and municipal hot spots, passed 90% confidence in three hot spots and passed 95% confidence in Chongqing Cold Point. (3) In 2018, the nearest neighbor ratio is 0.531149 (type: Clustered), Moran’s I is 0.083485, except Qinghai province, all other provinces held marathons. The Yangtze core circle has expanded to the whole East China area and was still expanding to North China, Central China, South China and even the Southwestern areas. East China, North China, Central Region and other eight provinces and cities hot spots passed 95% confidence, four hot spots passed 90% confidence, Tibet Autonomous Region cold spot passed 90% confidence. (4) By analyzing and discussion the spatial information, this paper divides the temporal and spatial evolution of China marathons into three stages. The first stage is the starting stage from 1981 to 2009, when the major influential factor was economic factor and minor factors were political and social ones. The second stage is the developing stage from 2010 to 2014, when Chinese society started to transform and the major influential factor was social factor. The third stage is the growing stage from 2015 to now, when political, economic and social factors work collectively.

The number of Chinese marathon events has been increasing rapidly year by year, from the decentralized holdings in 2010 to recent clustering and the holding places are mainly concentrated in East China, North China, Central China and South China. Its spatial distribution pattern is consistent with China’s economic and population divisions. There are more hosted events in the eastern region than in the western region and more hosted events in the southern region than in the northern region, with clear regional differences. The Yangtze River Delta, Beijing-Tianjin-Hebei, Pearl River Delta and Chengdu-Chongqing regions are the most intensive. According to the evolution of the marathon spatial distribution, it is found that economic, political and social factors affect the distribution pattern of marathon events. The past 38 years of development of the Chinese marathon, was divided into three stages due to different economic, political and social environments. This suggests that the marathon may be an inevitable result of people’s pursuit of higher level health needs after social and economic development has reached a high level. 

As seen from the conclusion, Chinese marathon industry has already entered a rapid growth stage. It is worth studying further, whether we should continue to expand and what the capacity of each city is. Also, as the Chinese marathon industry started relatively late, many events are still young and unstable. As a result, the micro changing pattern might be neglected in the research. When analyzing the influential factors for the spatial evolution of marathons in China, as the length of the paper is limited, this research fails to study the spatial distribution evolution of regional marathons. Also, when discussing the influential factors, it fails to quantify the policy, economy and society. For example, social factors can be quantified as population base, social development level, residence’s awareness of entertaining and the fitness level of the mass. The quantitation of these factors should be further studied. 

## Figures and Tables

**Table 1:** Cities that held more than 5 marathons/number of events/4 province tiers by the number of marathons.

Number of Events	City	Number of Events	City
12	Beijing	8	Wuxi/Wuhan/Chongqing
10	Nanjing	7	Shanghai
9	Chengdu/Suzhou/Zhengzhou	6	Hangzhou/Kunming/Qingdao, Shenzhen
**4 province tiers by the number of marathons**			
T-1	Jiangsu		
T-2	Shandong, Henan, Anhui, Hubei, Zhejiang, Guangdong, Sichuan, Yunnan		
T-3	Hebei, Henan, Shaanxi, Jiangxi, Guangzhou, Fujian, Hainan, Heilongjiang, Jilin, Hunan, Inner Mongolia, Shanxi, Guizhou		
T-4	Xinjiang, Tibet, Ningxia, Liaoning		

**Table 2:** Average nearest neighbor distance in 2010, 2015 and 2018.

Year	ANND (km)	Nearest Neighbor Ratio	*Z* Score	*p* Value	Type
2010	345.92	1.164714	1.336896	0.181257	Random
2015	66.60	0.502146	−10.817515	0.0000	Clustered
2018	61.28	0.531149	−16.587427	0.0000	Clustered

**Table 3:** Spatial autocorrelation in 2010, 2015 and 2018.

Year	Moran’s I index	*Z*-Value	*p* Value	*Z*-Value Test Results
2010	−0.010165	−0.541599	0.588095	non-significant
2015	0.066267	5.488973	0.000000	0.99
2018	0.083485	6.126412	0.000000	0.99

**Table 4:** Comparison of hot spot analysis between 2015 and 2018.

Years	Hot Spot 99% Confidence	Hot Spot 95% Confidence	Hot Spot 90% Confidence	Cold Spot 90% Confidence	Cold Spot 95% Confidence
2015		Jiangsu, Anhui, Henan, Liaoning	Beijing, Tianjin, Hebei		Chongqing
2018	Anhui	Jiangxi, Hubei, Henan, Shaanxi, Shandong, Jiangsu, Shanghai, Zhejiang	Shaanxi, Beijing, Tianjin, Hebei	Tibet	
